# Preparation and Characterisation of Waste Poultry Feathers Composite Fibreboards

**DOI:** 10.3390/ma13214964

**Published:** 2020-11-04

**Authors:** Riko Šafarič, Lidija Fras Zemljič, Miroslav Novak, Bogdan Dugonik, Božidar Bratina, Nenad Gubeljak, Silvester Bolka, Simona Strnad

**Affiliations:** 1 Faculty of Electrical Engineering and Computer Science, University of Maribor, Koroška cesta, Center 46, 2000 Maribor, Slovenia; riko.safaric@um.si (R.Š.); bogdan.dugonik@um.si (B.D.); 2 Faculty of Mechanical Engineering, University of Maribor, Smetanova ulica 17, 2000 Maribor, Slovenia; lidija.fras@um.si (L.F.Z.); nenad.gubeljak@um.si (N.G.); simona.strnad@um.si (S.S.); 3 Higher Vocational College Wood Technology School, Lesarska ulica 2, 2000 Maribor, Slovenia; miroslav.novak2@gmail.com; 4 Faculty of Polymer Technology, Ozare 19, 2380 Slovenj Gradec, Slovenia; silvester.bolka@ftpo.eu

**Keywords:** fibreboard, composites, poultry feathers, wood residues, construction material

## Abstract

The growth of poultry meat production is increasing industrial waste quantities every year. Feathers represent a huge part of the waste, and international directives and restrictions prevent landfilling of such biodegradable materials with high burning values. Furthermore, with their unique properties, poultry waste feathers are already a reliable resource for many byproducts, such as keratin extraction, fibres, hydrogel production, etc., all trying to achieve a high-added value. However, mass reduction of waste feathers into useful applications, such as development of alternative building materials, is also an important aspect. To take advantage of feathers’ thermal insulation capabilities, sound damping, and biodegradability, we worked towards mixing waste feathers with wood residues (wood shavings, dust, and mixed residues) for production of composite fibreboards, comparable to the market’s medium density fibreboards. The emphasis was to evaluate waste poultry feathers as the component of natural insulation composites, along with mixed waste wood residues, to improve their mechanical properties. Various composite fibreboards with different shares of wood and feathers were produced and tested for mechanical, thermal, and acoustic properties, and biodegradability, with comparison to typical particle boards on the market. The addition of waste feather fibres into the fibreboards’ structure improved thermal insulation properties, and the biodegradability of fibreboards, but decreased their bending strength. The sound transition acoustic loss results of the presented combination fibreboards with added feathers improved at mid and high frequencies. Finally, production costs are estimated based on small scale laboratory experiments of feather processing (cleaning and drying), with the assumption of cost reduction in cases of large industrial application.

## 1. Introduction

The poultry industry is undergoing strong expansion globally. According to the Food and Agriculture Organisation, in 2015 global production of poultry meat amounted to 100.6 million tons, and the forecast for 2030 is 143.3 million tons [1]. In the EU-28, 13 million tons of poultry meat was produced in 2014, which was about 9% more than in 2007 [2]. One of the main waste types of this food industry sector are feathers. It was estimated that about 8–9 Mt of feathers are generated in the world every year [3]. The EU Directive 1999/31/EC on the landfill of waste [4] restricts the landfilling of disposals that contain large quantities of biodegradable materials with high burning values. Therefore, finding the right and effective way to reuse waste poultry feathers will be one of the most important achievements in the field of industrial waste management and recycling in the future. The world practice for poultry feather waste management is sanitation and land-filling, and the costs for such disposal are borne by the poultry industry, and, finally, by consumers [5,6]. Currently, feathers are also converted into feather meal for low nutritional value pet food, which is exported mainly to Eastern Europe and Russia, However, these are only partial and low-cost solutions, as only a small part of the total waste volume was used for this purpose. Possibilities are, therefore, being sought to use poultry feather wastes in higher value products, and solutions where the consumption of waste feathers would be much more significant.

Poultry feathers are one of the most ubiquitous and inexpensive agricultural byproducts available in the world. The most important properties, like low density, high flexibility and compressibility, high warmth retention, and the ability to dampen sound, provide them unique properties unlike any other natural or synthetic fibrous material [7]. Furthermore, feathers represent a large potential source of keratin, as they are about 90% composed of this structural protein [8].

Many attempts have been made during recent decades, to evaluate the potential of waste feathers in higher added value applications. Natural and chemically treated poultry feathers were tested for their ability to absorb/remove different heavy metals like copper, zinc, calcium, magnesium, iron, and manganese from industrial wastewaters [9,10]. Poultry feathers were investigated as waste material for the development of a new low-cost hydrogen storage substrate [11]. In another investigation, authors investigated pyrolyzed feathers’ application for catalytic carbon production [12,13,14] and in biobased composite reinforcements [15]. However, the production processes of these materials require a large input of chemicals, energy, and time to clean and decompose the waste feathers. In papers [16,17] the authors reported on the preparation of composites containing poultry feathers, cotton linters, and PET or PP fibres using a paper-making method. The authors suggested applications such as paper-like products in artistic painting or composite mats for technical oil removal. Recent publications on the waste feathers’ application for composite materials development report mainly on the combination of waste feathers with synthetic polymers such as polylactide, polyethylene, epoxy-resin, polyurethane, etc. [5,18,19,20,21,22,23]. In all these investigations synthetic polymers represent one of the main components, and the proportion of waste feathers in composite materials is rather low (below 10%). Another important area of research deals with the use of keratin, extracted from waste poultry feathers, and the development of a variety of keratin materials, structures, and products, such as films, fibres, or hydrogels for different fields of application, like medicine, water cleaning, energy-saving, electronics, food packaging, and graphene production [24,25,26,27]. However, the main disadvantage of extracting keratin from feathers is still the ecologically burdensome chemical extraction process and the relatively low average molecular weight of keratin from feathers. Despite an extremely extensive search for the possibilities of new applications, the effective conversion of large quantities of poultry feather waste into products with higher added value is still pending.

One of the most appealing ideas for poultry feather waste applications in larger amounts is the development of alternative building materials. Composite building insulation materials could, potentially, consume large amounts of waste poultry feathers, and, therefore, any new findings and developments in this field could be of great importance [28]. Commonly, building insulation is realised using materials obtained from petrochemicals (mainly polystyrene), or natural sources processed with high-energy consumptions (glass and rock wools). The introduction of the concept of sustainability in the building design process encouraged researchers aimed at developing thermal and acoustic insulating materials using natural or recycled materials. However, the use of alternative materials for insulating building materials is not particularly widespread, since, in 2011, mineral wool and plastics still represented 52% and 41% of the market share. The majority of applications of unconventional green and/or recycled materials for building insulation is limited to a prototypal or experimental and laboratory stage. As alternative building insulation materials different plants or plant parts (reeds, bagasse, cattail, corn cob, cotton stalks, etc.) have been investigated, and recycled materials (glass foam and fibres, plastics, textile fibres, and textiles, etc.) [28]. Poultry feathers have been investigated as reinforcements in cement-bonded composites [29], as well as a component of medium density fibreboard composites [30,31]. In both cases, the addition of poultry feathers in amounts higher than 10% influenced the lowering of mechanical properties but increased the water repellence of the prepared composite materials. The medium density fibreboards were made from conventional woods such as aspen, beech, and poplar, using conventional production methods. Poultry feathers were added only in smaller amounts as an alternative component (5%–10%). The authors found out that wollastonite, which was added to urea-formaldehyde resin, acted as a reinforcing filler, which improved the mechanical properties of fibreboards. The authors in [32] made the characterisation of the fibreboard made by only poultry feathers (60%–80%) and adhesive epoxy resin (20%–40%). Our method used only 10% of adhesive, which made it more economically appropriate and less burdensome for the environment in the case of biodegradability.

The main objective of this study was to evaluate the use of poultry feather waste, together with wood waste from various sources, as a component for the production of natural insulation composite fibreboards. The fibreboard composites were prepared using different amounts of poultry feather waste (20%–70%) and wood waste in the form of wood shavings or mixed wood residues. Their mechanical, thermal, and acoustic properties were investigated and discussed, and their biodegradability.

## 2. Materials

### 2.1. Poultry Feathers

Poultry feathers were obtained from a poultry meat production company (Perutnina Ptuj, Slovenia), in the form of the final waste in the production and processing of poultry meat. Feathers were cleaned industrially, and washed on a grid with running water, which removed large impurities, such as secretions, blood, etc.

### 2.2. Wood Residues

**Figure 1:**
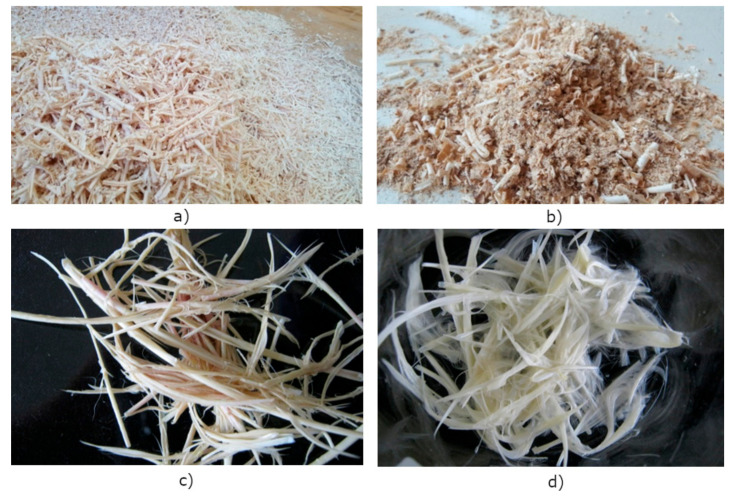
Waste residues: (**a**) wood shavings; (**b**) mixed wood residues; (**c**) waste poultry feathers after industrial precleaning; and (**d**) feathers after the laboratory washing and rinsing process.

The wood residues used in the manufacturing process of sample fibreboards were a byproduct of seasoned wood processing. Two types of wood residues were used: Shavings and mixed wood residues. Shavings were made in the processing of solid wood of different tree species in the planer (Figure 1a). Mixed wood residues were produced in the technological process of solid wood products from different tree species or other wooden semi-finished products (different types of wooden boards, etc.). The mixed wood residues consisted of sawdust, shavings, various cuttings of milling, boring and turning machines, and wood dust (Figure 1b).

### 2.3. Adhesive

Melamine-urea-formaldehyde adhesive MELDUR H 97 with an emission class E1, purchased from the company Melamin Kočevje, Slovenia, was applied as an adhesive component in the sample fibreboards.

## 3. Methods

### 3.1. Waste Poultry Feather Cleaning

Before application, the industrially precleaned waste feathers (Figure 1c) were inserted into a mesh bag and washed in a commercial washing machine at 60 °C with the addition of non-ionic detergent (Sandoclean PC, Sandoz). The optimised cleaning procedure was defined in the initial studies and is described in detail elsewhere [33]. Cleaned feathers after the laboratory washing and rinsing process are shown in Figure 1d.

### 3.2. Waste Poultry Feather Drying

After the cleaning process, feathers contained approximately 75% of water. The drying procedure had to be performed very carefully, because wet feathers are easy to be polluted by fungus spores from the air. Furthermore, care had to be taken that drying temperatures were low enough and did not change the physical or chemical structure of the feathers. Therefore, a modified vacuum drying method, developed primarily for drying of sludge (see Supplementary Material, Figure S1), was applied for controlled drying of feather samples. The method is based on the physical effect that water evaporates from the feathers at temperatures between 36 and 42 °C if the pressure in the vacuum chamber is lower than 80 mbar. Warming up the vacuum chamber was performed by the waste warm water from industrial processes. Furthermore, the low pressure affected the additional decrease of bacterial and fungus flora and fauna up to ten times [34].

**Figure 2:**
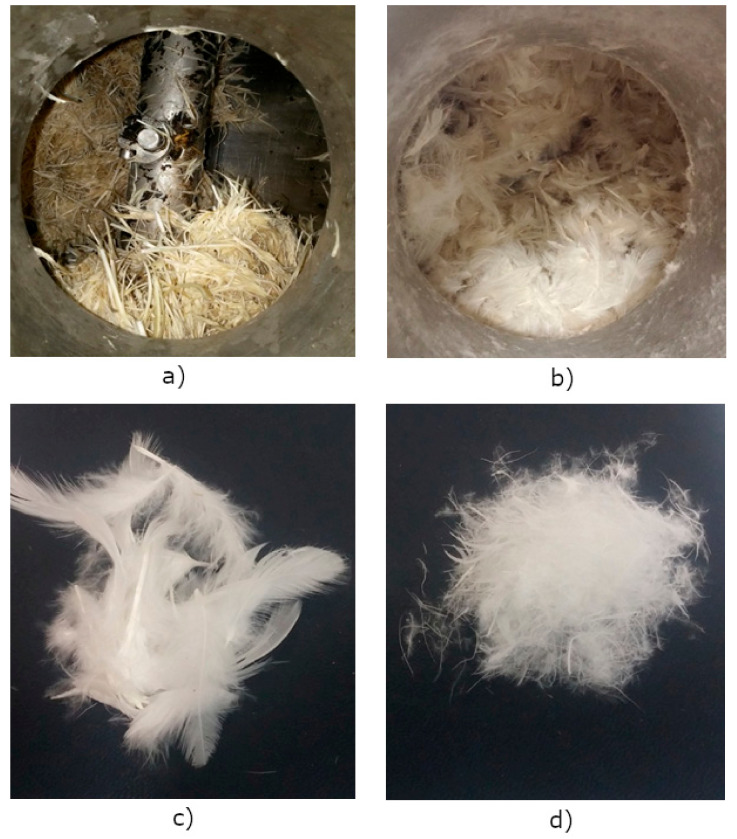
Volume increases inside of the drum drier: (**a**) initial wet feathers before drying; (**b**) completely dry feathers after drying, (**c**) dried feathers; and (**d**) shredded/dried feathers.

The original drying process had to be modified, since the drying of feathers differs from sludge drying. The biggest difference is in the materials’ volume change. The total volume of feathers increases with drying (fluffiness, see Figure 2a,b), whilst sludge drying reduces the total volume as the water evaporates (grains). Contact drying demands stirring to achieve adequate temperature distribution, therefore a drum-shaped drier was used. The blades were set up properly and designed to prevent feather lump formation under the blades, which could get stuck and cause ineffective drying. Therefore, adequate stirring control and speed adjustment were needed, and changing the stirring direction periodically. After 24 h of drying the remaining water in the feathers was decreased from approximately 75% to 17%. After an additional 5–10 h of drying, 10% of moisture content in feathers was reached. Such dried feathers had a neutral or typical dry feathers smell. The pH of the evaporated and condensed water amounted to around 10.

**Figure 3:**
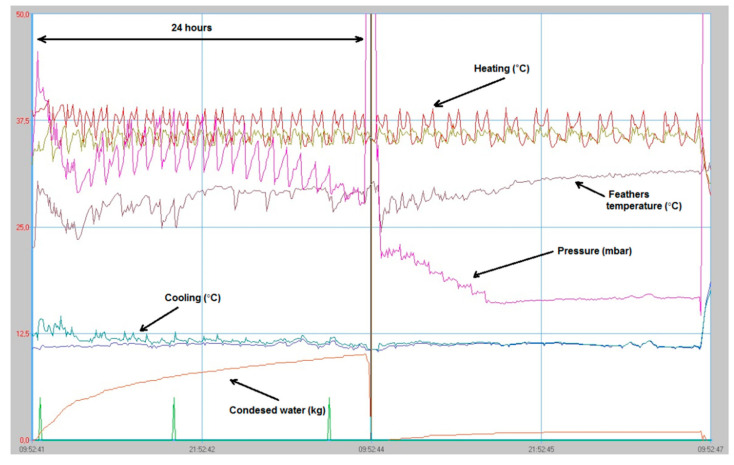
The measurement variables vs. time (pressure, the temperature of the dried material, temperature of the heating and cooling water, and the weight of condensed water).

Figure 3 presents the measured values of the drying process in the vacuum chamber (chamber, feathers, and cooling water temperatures, chamber pressure, and condensed water mass). The temperature of the input warm water was between 36 and 42 °C, the cooling water needed for the water condensation part of the process was between 10 and 13 °C, and the pressure in the chamber decreased from an initial 45 mbar to a final 14 mbar. The temperature of the feathers was between 24 and 35 °C. The initial wet poultry feathers (15 kg) produced approximately 10 kg of condensed water in the process after 24 h, and 1 kg more after the next 10 h, and produced the final 3.6 kg of dried feathers with 10% of moisture. The rest of the water was lost in the process through the vacuum pump. Such dried feathers were prepared for long term indoor storage. Even after 2 years of indoor storage, the dried feathers were intact, due to biodegradation, with a still neutral smell. The dried feathers (Figure 2c) were ready to be used for the production of the wood-feather fibreboards. Figure 2d presents the shredded and dried feathers, which were not used in our experiment due to the expensive industrial shredding procedure.

### 3.3. Fibreboards’ Preparation 

Preparation of fibreboards samples was performed in the wood workshop by mixing feathers with wood shavings or mixed wood residues. The boards with wood shavings had a coarse structure (denoted by C in Table 1), while the boards made with the mixed wood residues had a finer and denser structure (denoted by F in Table 1). The amount of added adhesive was the same for all the samples (10 wt %). The preparation was performed at room temperature *T* = 26 °C and relative air humidity *RH* = 54%. Fibreboard samples were made in dimensions 330 mm × 380 mm × 10 mm.

**Figure 4:**
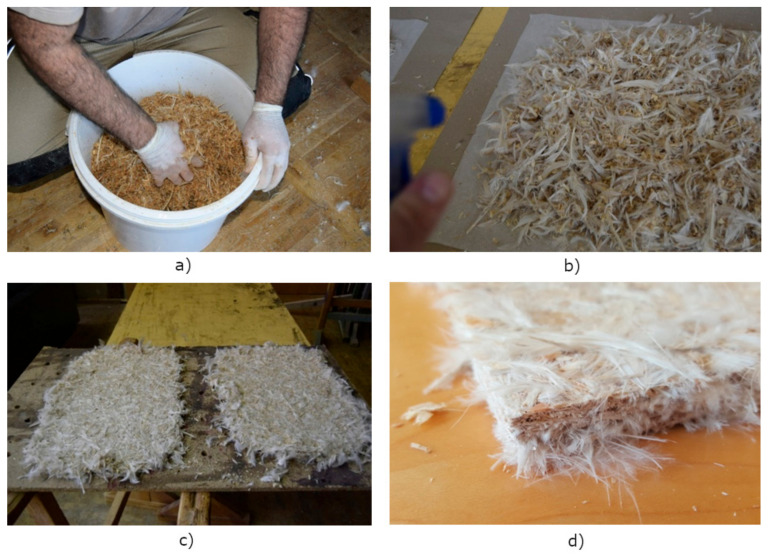
Preparation of fibreboards: (**a**) wood mixture; (**b**) mixture of feathers and wood shavings; (**c**) fibreboard samples after pressing; and (**d**) cut out fibreboards for testing.

The weighed quantities of feathers and wood wastes were mixed in a dry state until the uniform distribution was reached of the individual components. After that, the adhesive was added, using a spray method during simultaneous mixing. The final mixing of all components was performed manually (Figure 4a,b).

Pressing of the sample boards was done in a two-stage hydraulic press at the temperature *T* = 60 °C and pressure *p* = 50 bar. Spacer bars of 10 mm thickness were applied to provide the same final thickness of the boards. The prepared plates were cooled down for a few hours and then conditioned for a few days at the defined temperature (*T* = 20 °C) and humidity (*RH* = 40%; Figure 4c,d). To achieve better dimensional stability, the prepared feathers/wood plates were placed between two plywood layers (1.5 mm thick). Sandwich structures were denoted with S. The sample’s compositions and denotations are listed in Table 1.

Sample fibreboards were weighed and measured to determine the density of each fibreboard combination. According to Standards, the density fits them between the low density fibreboard (LDF) with density 0–650 kg/m^3^ (type F, SC(70/20), and SF(20/70)), and medium density fibreboard (MDF), with density 650–800 kg/m^3^ (type SF(70/20) and SC(20/70)). High-density fibreboards (HDFs) had densities higher than 800 kg/m^3^. Calculated densities are shown in the rightest column of Table 1.

### 3.4. Mechanical Testing 

To determine the strength of fibreboards samples, three-point bending tests were performed by using the servo-hydraulic testing machine INSTRON 1255 (see Supplementary Material, Figure S2).

Tests were performed at room temperature 23.5 °C and humidity 56% in displacement control with piston velocity 10 mm/min by Instron Viewmaker software and control unit 8500+. All specimens had the same sizes. For all tests, the same span distance of 130 mm and the same pins were used as shown in Supplementary Material Figure S2. The load–deflection points were recorded during the tests. All tests were terminated after reaching the maximum sustained load. The points for stress–deflection curves were calculated based on theoretical equations for bending strength.

### 3.5. Acoustic Analyses

The main purpose of the acoustical part of the study was to determine the sound transmission loss (STL) of the given materials as the proportions of a mixture of the feathers and pieces of wood. The acoustical measurements determine the loss of amount in decibels (dB) owing to the transmission through the material. Different techniques exist to determine the acoustic properties of materials. Mostly, the impedance tube method (ITM) [35,36,37], or intensity room method (IRM) [38,39] are applied for measuring STL. All these measurement techniques are based on the determination of the sound pressure at specific locations.

A source and a receiving room were applied in the intensity room method. A random noise sound generator and powered loudspeaker were used to produce a diffuse sound field in the source room. Test material is placed between both chambers. The sound field in both rooms was measured by the same type of microphones. The difference in sound intensity in the source and receiving room is defined as STL.

**Figure 5:**
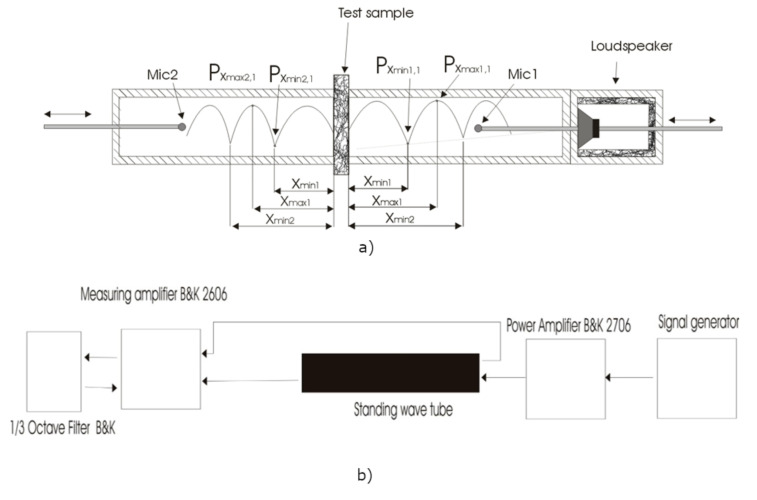
Measuring setup: (**a**) standing wave tube and (**b**) measuring arrangement for sound transmission loss measurements.

Alternatively, the impedance tube method was applied for measuring STL [22], where only small samples were needed of the absorbing material. A sound source (loudspeaker) was mounted at one end of the impedance reverberant source tube, and a sample of the material was placed at the other end, as illustrated in Figure 5a. The loudspeaker generated sound waves of different frequencies, which propagated as plane waves in the tube, striking the sample mounted on the end of the source tube. A part of the waves reflected into the source tube, a part was absorbed by the material, and a part passed through the material to the receiving tube. The test sample being measured was placed between two tubes of 10 cm in diameter. The joints between tubes and samples were sealed with rubber materials to avoid sound leakage. The receiving tube was terminated with a reflective material. 

In tubes, a standing wave was produced, and the first maxima and minima (*P*_xmax_ and *P*_xmin_) were then measured by movable microphones (Mic1 and Mic2) installed on the carriage. To control the accuracy of the generated standing frequency, the distance was measured between two successive minima or maxima (*X*_min_ and *X*_max_). By measuring, the sound pressure at four measuring points (maxima and minima) is defined by moving the microphone in the impedance tube (in the source tube and the receiving tube) to determine the transmission loss of the material. The sound pressure level was measured for both upstream and downstream tubes on one-third octave frequency bands between 250 Hz and 4 kHz. The results of the transmission loss of an individual testing probe were analysed. Measurements in the tube (see Figure 5b) were made using ½” B&K Condenser Microphones Type 4134, which were supplied by a Microphone Amplifier Type 4204. Microphones were calibrated by a B&K 4220 Microphone Calibrator. The broadband speaker in the tube was driven by a B&K Power Amplifier 2706. The testing sinusoidal signal was generated by Arta software (version 1.9.0). The signal from the microphone was measured by a B&K Measuring Amplifier Type 2606. The B&K microphones and amplifiers are from the manufacturer Brüel & Kjær, Nærum, Denmark. To avoid distortion of the measured results caused by environment noises, the measurements were performed in a semi-reverberant room.

### 3.6. Biodegradation Measurement (Based on ISO 14855-2)

A biodegradation test was performed using the ECHO RepirometerTyp ST 4+ (Echo, Slovenske Konjice, Slovenia). All measurements were performed in controlled compost at 58 °C and 50% humidity. The airflow was set to 0.2 L/min over 210 days. Six reactors were used; the first one with compost as a reference, the other 5 with samples of biodegradable material and compost; 50 g biodegradable material; and 327 g of compost. The CO_2_ amounts produced were measured 9 times a day. The produced amount of CO_2_ of the biodegradable samples was subtracted from the reference amount of produced CO_2_ (the data from the reactor with only compost). Once a week, the biodegradable samples and the compost were well mixed, and the water content was controlled. All the samples were cut in a cuboid shape with dimensions less than 10 mm.

### 3.7. Thermal Conductivity Determination

The thermal conductivity test was performed using a Hot Dick type TPS 1500 device from Hot Disk, Göteborg, Sweden. For all measurements, the Kapton Dick type from Hot Disk, Göteborg, Sweden was used, with a diameter of 6.403 mm. The power was set to 10 mW, and the measuring time was set to 160 s. The measurements were performed at 21 °C. All measurements were performed with two samples with a sensor in between. The test is based on transient plane source thermal characterisation, where the thermal conductivity is defined as the amount of heat per unit time and unit area that can be conducted through a plate of unit thickness.

## 4. Results and Discussion

### 4.1. Structure and Morphology of Samples

**Figure 6:**
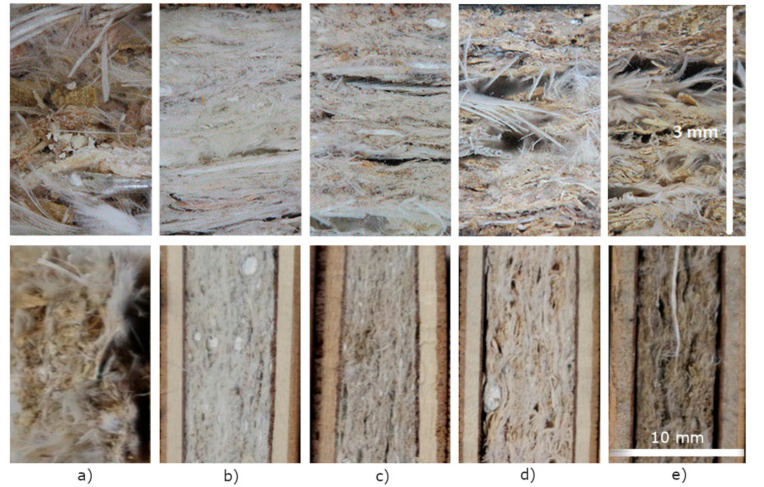
Horizontal close look and cross-sections of fibreboard samples: (**a**) blend structure F(70/20); (**b**) fine sandwich structure SF(70/20); (**c**) fine sandwich structure (c) SF(20/70), (**d**) coarse sandwich structures SC(70/20); and (**e**) coarse sandwich structures SC(20/70).

Prepared fibreboard samples had different morphology, depending on the average size of the ingredients’ particles (Figure 6). The macro image view of samples was made with the DermLite DL4 Dermatoscope from 3Gen Inc., San Juan Capistrano, CA, USA which was attached to the lens of a Canon M10 photo camera. The upper row images in Figure 6 present horizontal close look of the composite fibreboards, whereas the lower row shows their cross-section.

Figure 6b–e shows the sandwich structure of mechanically reinforced samples with plywood layers (width was 1.5 mm) on both sides. There were significant differences between the fine and coarse structures of the samples. Wood residues, which consisted of residues from different wood processing phases and were mostly in the form of fine wood particles or dust, enabled the preparation of fine and uniform mixtures with feathers. Therefore, these samples had featureless structures without any larger holes or empty regions. On the contrary, larger holes and empty places could be observed in the structures of the samples prepared by wood shavings. The structure of the blend feather/mixed wood residues, plate F, differed significantly from the other four sandwich structures, thus, owing to the less compact structure, cutting was difficult and uneven, and large amounts of feathers projected from the surface.

### 4.2. Mechanical Properties

**Figure 7:**
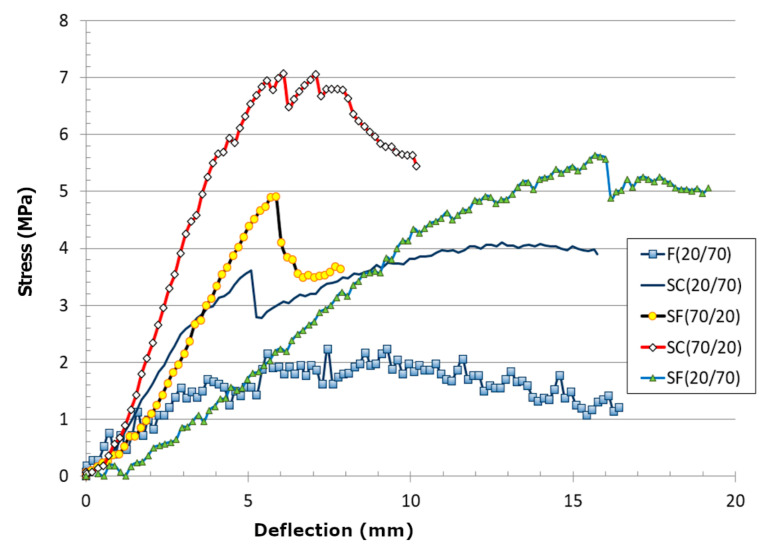
Stress–deflection curves as the results of the three-point bending testing of fibreboard samples.

The stress–deflection curves of fibreboard samples are presented in Figure 7. Based on theoretical equations for bending strength, the points for stress–deflection curves were calculated and provided graphically, as is shown in Figure 7. The results showed the highest bending strength for the coarse sandwich structured fibreboard with a lower share of feathers (SC (20/70)). This sample showed the longest linear loading parts with few “pop-ins” and the highest Young’s modulus as well. Surprisingly, the samples with higher amounts of feathers (SC (70/20) and SF (70/20)) showed the second-highest moduli among all. However, on the other hand, the samples with high amounts of feathers demonstrated lower maximum bending strengths of about 5 MPa and 3.5 MPa respectively. The lowest maximum bending strength was determined for the blend sample F (about 2 MPa). As expected, the increase in feather shares reduced the bending strength of the material. However, all samples showed unstable loading behaviour as a consequence of heterogeneous materials’ composition and orientation. One should keep in mind that bending presents a more critical manner of loading than tensile loading. Therefore, it is possible to consider the obtained bending strength as the maximum bending stress of samples for the component’s design. For comparison, maximum bending strength values of equivalent size wooden particleboards (chipboards) were between 11 and 14 MPa, depending on the board type and thickness. 

### 4.3. Acoustic Properties

**Figure 8:**
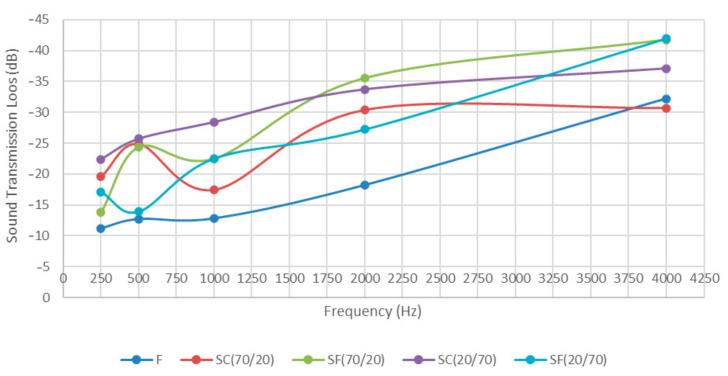
Sound transmission loss (STL) for different fibreboard samples.

Transmission loss measuring was performed on five different material samples. Generally, in compliance with the rule of thumb, the highest was the material’s density (mass law) and the better one was its sound insulation. This rule is especially true for low sound frequencies. Porous materials allow airflow and are generally less effective as STL barriers. Certainly, porous materials placed inside wall cavities generally improve sound insulation. The sound transmission loss in decibels (dB) on one-third octave frequency bands between 250 Hz to 4 kHz for each fibreboard sample is presented in Figure 8. As expected, the lowest STL in the frequencies below 3.5 kHz, was determined for sample F, which, only at the highest frequencies of 4 kHz, showed slightly better results than the sandwich sample with the coarse structure and the highest amount of feathers SC 70/20. At mid-tone frequencies (2 kHz), the highest STL was achieved by sample SF 70/20, and the difference between this sample and sample F was around 18 dB. At higher frequencies, both samples with a fine structure (SF 70/20 and SF 20/70) showed the best STL, which confirmed the basic theory of sound transmission. The density of samples had the major influence on STL, and not so much the feather/wood composition. In comparison to the presented feather/wood fibreboards, a typical wooden particleboard showed the best STL result at low frequencies, while STL results of the presented combinations improved at mid and high frequencies (2 kHz and 4 kHz). The results agreed well with the results of acoustic analyses of similar materials [32,40].

### 4.4. Biodegradation

**Figure 9:**
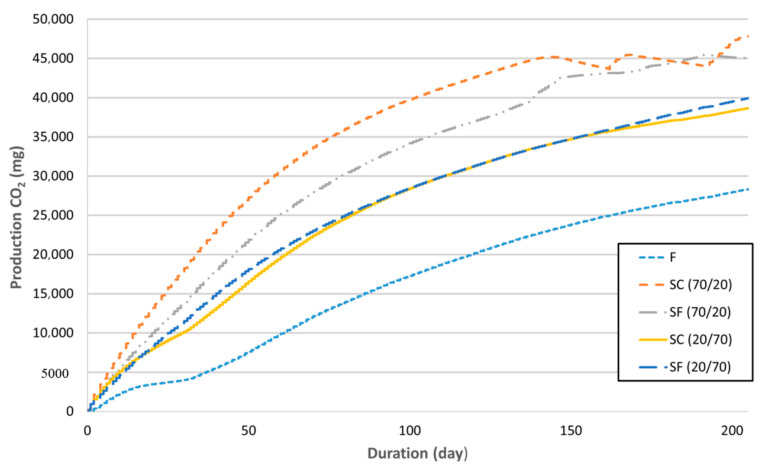
Biodegradation of the samples as a function of CO_2_ production.

In Figure 9 the biodegradation is presented as a function of CO_2_ production from the biodegradable samples already subtracted from a pure compost CO_2_ production. The slowest biodegradation occurred in the sample with the blend structure (F), followed by the samples SC(20/70) and SF(20/70). Samples SC(70/20) and SF(70/20) showed the quickest biodegradation, with the highest amounts of feathers in the compositions. Furthermore, the coarse structure of fibreboards influenced quicker biodegradation, thus the sample SC(70/20) showed, in 210 days of measuring time, the quickest biodegradation in comparison to SF(70/20) and other samples. The coarse structured fibreboard with the low amount of poultry feather SC(20/70) showed a slightly different biodegradation timeline in comparison to the fine structured fibreboard with the same composition SF(20/70). In the first 18 days, SC(20/70) degraded quicker, in the following approximately 99 days slower, then, in the next 158 days the degradation of both samples ran in the same way, and from day 158 to 210, again, slower biodegradation of the sample SC(20/70) was observed. 

In general, the coarse structured fibreboards degraded quicker, most probably owing to better surface contact of the ingredients with the compost and the air. The degradation of the fibreboards was also accelerated by the higher amounts of poultry feathers in the composition. After 210 days, the biodegradation of the samples with 70/20 composition was almost completed, while the biodegradation of the samples with 20/70 composition blend (F) and sandwich structure (SC and SF) was slower by about 40% and 15% respectively. Assuming the biodegradation time of a typical wood residue particleboard was much longer, it was, hence, not included in the experiments.

### 4.5. Thermal Conductivity

The results of the thermal conductivity measurements are presented in Table 2. 

Due to the measurements principle, poultry feathers had the highest thermal conductivity. For the measurements, the poultry feathers had to be pressed together to achieve good surface contact between the sample and the sensor. Sample SC(70/20) showed the lowest thermal conductivity the followed by samples SF(70/20), SC(20/70), and SF(20/70). As expected, the coarse fibreboard structure, in combination with a higher content of poultry feathers, showed lower thermal conductivity compared to the finely structured wood wastes and poultry feathers’ fibreboards. Poultry feathers, as a great thermal insulation material, and the structure with larger holes and pores with lots of entrapped air, enabled the highest thermal insulation, i.e., the lowest thermal conductivity of 0.11 W/mK. In the case of thermal insulation, the presented feather/wood fibreboards all achieved better results against typical wood particle boards, where typical thermal conductivity is between 0.17 and 0.21 W/mK [32].

### 4.6. Additional Costs to Fibreboards’ Production 

The proposed preparation of waste poultry feathers for composite fibreboards needs adequate washing and drying steps. Costs for both are determined by laboratory experiments and market prices. Investments in infrastructure and amortisation are not taken into consideration. Additionally, due to the specific volume of dried feathers, it is more convenient that the infrastructure needed to produce fibreboards should be placed near a poultry production facility to avoid unnecessary storage and transportation costs.

The estimated cost of feather washing was based on a typical washing machine’s consumption and detergent prices. As mentioned, industrially precleaned waste feathers (with 70%–80% of water volume) were washed at 60 °C, with the addition of non-ionic detergent. By laboratory experiments, the cost of washing cycle consumption (electricity, water, and detergent) was estimated as 0.55 €/kg of dry feathers. Such washed feathers need to be dried to reduce the water to values between 10% and 20% of water volume prior to composite fibreboards’ production. Drying was based on a vacuum technology procedure, where feathers were dried for 24 h to decrease the water volume to 20%, and an additional 10 h to decrease it to 10%. The cost of drying was determined by Standard EN 1434-1: 2006, which calculates the consumed energy (heating, evaporation) for the drying process. It was calculated that approximately 0.8 kWh of heat energy was needed to evaporate 1 kg of water out of wet feathers. According to market energy prices and the amount of dried feathers in the experiments, the calculated cost for the first case was 0.26 €/kg of dry feathers (feathers with 20% of water volume), and for the second case 0.28 €/kg of dry feathers (10% of water volume). Total costs of washing and drying were, hence, between 0.81 and 0.83 €/kg of dry feathers for the first and second cases, respectively.

Experimental production of various composite fibreboards determined the necessary volume of feathers in one square metre (or calculated to a cubic metre) of fibreboard, depending on board type and the mixture of feathers, wood shavings, or residues. Which type of dry feathers (10% or 20% of water volume) is more suitable for fibreboard production depends on thermal, mechanical, and/or acoustic demands. Market prices for LDF were around 165 €/m^3^ and for MDF around 235 €/m^3^. With the added amount of dry feathers into various combinations of fibreboards, the estimated price for F, SC(20/70), and SF(20/70) would be between 180 and 280 €/m^3^ and for SF(70/20) and SC(70/20) between 430 and 450 €/m^3^.

The calculated additional costs of washing and drying into the fibreboard production, the proposed fibreboards market prices would rise by approximately 10%, 20%, and 40%, in the case of F, SC(20/70), and SF(20/70) combinations, respectfully, which used less volume of dry feathers, and approximately 90% and 150% in the case of SF(70/20) and SC(70/20) combinations, respectfully, which used a higher volume of dry feathers. For the first category of fibreboards with a smaller price increase, the price could be justified with more environment-friendly, improved isolation, and acoustic benefits towards classic ones; however, this is questionable for the latter proposed fibreboards with a higher market price increase. Finally, the proposed cost estimations were based on small laboratory scale equipment; hence, using industrial large-scale equipment the costs would be reduced considerably.

## 5. Conclusions

In this research, waste poultry feathers were applied, together with wood residues, for the production of fibreboard samples, in order to analyse the possibilities of reusage of larger amounts of these wastes for value-added products. Such a combination of wastes with integration of an alternative modified vacuum drying process has not been investigated so far.

For that purpose, waste poultry feathers from the poultry meat industry were cleaned, dried, and mixed with different amounts and different wood residues, i.e., wood shavings and mixed wood residues. Two different structures of fibreboards were formed, blend (F), and sandwich structures (S), with a coarse (C) and fine (F) structure. 

The wood residue’s form and the preparation procedure influenced the fine or coarse structure of fibreboard samples significantly. Samples made of mixed wood residues had more even, finer, and denser structure, in comparison to the coarser structure of fibreboards made from wood shavings. The latter had larger empty regions, and pores visible in the sample’s cross-section’s micrographs.

Mechanical properties were evaluated by means of a three-point bending test. A coarsely ground fibreboard sample with 20% feathers (SC(20/70)) showed the highest bending strength. This sample also showed the longest linear loading parts with few “pop-ins”. The lowest bending strength was determined for the sample with a blended structure of feathers and wood residues (F(20/70)). As it was expected, the higher shares of feathers in the fibreboard structures reduced (by about 20%) the bending strength of the material, while the fine or coarse structure of the boards did not have any significant influence on bending strengths.

On the other hand, the acoustic properties of fibreboards were improved mostly by the different structural density of samples, and not so much by the material’s composition. As expected, higher sound transmission loss (STL) was demonstrated in the case of both denser packed fibreboard samples (SF (70/20) and SF (20/70)). Higher amounts of feathers in the fibreboards significantly increased their thermal insulation properties and their biodegradability. The thermal conductivity of samples with 70% of feathers was about 20% lower than that of the samples with only 20% feathers in the composition. As expected, the acoustic properties of fibreboards depended mostly on the structural density of the fibreboards and the feather proportion did not have a significant influence. The results of this research showed that also with the combination of two different natural waste materials, poultry feathers and wood residues, composite fibreboards could be designed with good thermal insulation capabilities and biodegradability. In addition, the combination of poultry feathers with amounts (20%–70%) and wood residues in the production of fibreboards made this method potentially attractive to use a high quantity of poultry feather waste and produced the building material, which in some technical aspects outperformed the classical wood fibreboards.

## Figures and Tables

**Table 1:** Denotation and composition of fibreboard samples.

Sample Denotation	Description	Feather Share (%)	Waste Wood Share (%)	Adhesive Share (%)	Density (kg/m^3^)
F	Blend Structure (Feathers/Mixed Wood Residues)	20	70	10	485
SC(70/20)	Sandwich Structure—Coarse (Feathers/Wood Shavings)	70	20	10	632
SF(70/20)	Sandwich Structure—Fine (Feathers/Mixed Wood Residues)	70	20	10	653
SC(20/70)	Sandwich Structure—Coarse (Feathers/Wood Shavings)	20	70	10	734
SF(20/70)	Sandwich Structure—Fine (Feathers/Mixed Wood Residues)	20	70	10	619

**Table 2:** Thermal conductivity (λ (W/mK)) of the fibreboard samples.

	F	SC(70/20)	SF(70/20)	SC(20/70)	SF(20/70)
λ (W/mK)	0.18	0.11	0.13	0.14	0.15
STD (W/mK)	0.02	0.01	0.02	0.01	0.01

Remark: STD is the standard deviation.

## Supplementary Materials

The following are available online at https://www.mdpi.com/1996-1944/13/21/4964/s1, Figure S1: Vacuum drier, and at https://www.mdpi.com/1996-1944/13/21/4964/s2, Figure S2: The three-point bending test performance using the servo-hydraulic testing machine INSTRON 1255.

## References

[1] Bruinsma J. (2003). Food and Agriculture Organization (FAO). World Agriculture: Towards 2015/2030.

[2] Marquer P., Rabade T., Forti R. (2014). Meat production statistics - Statistics Explained, Eurostat. https://ec.europa.eu/eurostat/statistics-explained/index.php?title=Archive:Meat_production_statistics.

[3] Lasekan A., Abu Bakar F., Hashim D. (2013). Potential of chicken by-products as sources of useful biological resources. Waste Manag..

[4] Council Directive (1999). Council Directive 1999/31/EC on the landfill. Off. J. Eur. Communities.

[5] Dieckmann E., Eleftheriou K., Audic T., Lee K.Y., Sheldrick L., Cheeseman C.R. (2019). New sustainable materials from waste feathers: Properties of hot-pressed feather/cotton/bi-component fibre boards. Sustain. Mater. Technol..

[6] Staron P., Banach M., Kowalski Z., Staron A. Hydrolysis of Keratin materials derived from Poultry Industry. Proceedings of the ECOpole Conference.

[7] Reddy N. (2015). Non-food industrial applications of poultry feathers. Waste Manag..

[8] Esparza Y., Ullah A., Boluk Y., Wu J. (2017). Preparation and characterization of thermally crosslinked poly(vinyl alcohol)/feather keratin nanofiber scaffolds. Mater. Des..

[9] Al-Asheh S., Banat F., Al-Rousan D. (2003). Beneficial reuse of chicken feathers in removal of heavy metals from wastewater. J. Clean. Prod..

[10] Sayed S., Saleh S., Hasan E. (2005). Removal of some polluting metals from industrial water using chicken feathers. Desalination.

[11] Senoz E., Wool R.P. (2011). Hydrogen storage on pyrolyzed chicken feather fibers. Int. J. Hydrogen Energy.

[12] Rangaraj V.M., Edathil A.A., Kadirvelayutham P., Banat F., Vengatesan M.R. (2020). Chicken feathers as an intrinsic source to develop ZnS/carbon composite for Li-ion battery anode material. Mater. Chem. Phys..

[13] Tyagi A., Yadav A., Sinha P., Singh S., Paik P., Kar K.K. (2019). Chicken feather rachis: An improvement over feather fiber derived electrocatalyst for oxygen electroreduction. Appl. Surf. Sci..

[14] Veerakumar P., Salamalai K., Dhenadhayalan N., Lin K.C. (2019). Catalytic Activity of Bimetallic (Ruthenium/Palladium) Nano-alloy Decorated Porous Carbons Toward Reduction of Toxic Compounds. Chem. – Asian J..

[15] Senoz E., Stanzione J.F., Reno K.H., Wool R.P., Miller M.E.N. (2012). Pyrolyzed chicken feather fibers for biobased composite reinforcement. J. Appl. Polym. Sci..

[16] Wrześniewska-Tosik K., Marchut-Mikołajczyk O., Mik T., Wieczorek D., Pałczyńska M. (2012). Mats for removing technical oil contamination. Fibres Text. East. Eur..

[17] Wrześniewska-Tosik K., Marcinkowska M., Niekraszewi A., Dorota A., Potocka T., Mik M., Pałczyńska M. (2011). Fibrous composites based on keratin from chicken feathers. Fibres Text. East. Eur..

[18] Choudary R., Krishna N.N., Bhargava N. (2018). Study on CFF-Polyester Composites. Mater. Today: Proc..

[19] Choudary R., Nehanth R. (2019). Effects of fibre content on mechanical properties of chicken feather fibre/PP composites. Mater. Today Proc..

[20] Isiaka Oluwole O., Avwerosuoghene Moses O., Joseph Ajibade O., Moshibudi Caroline K. (2018). Evaluation of the mechanical properties of chemically modified chicken feather fibres reinforced high density polyethylene composites. J. Taibah Univ. Sci..

[21] Manral A.R.S., Gariya N., Bansal G., Singh H.P., Rawat A. (2020). Computational stress analysis of Chicken Feather Fibre (CFF) with Epoxy-Resin matrix composite material. Mater. Today: Proc..

[22] Sun M., Sun H., Hostler S., Schiraldi D.A. (2018). Effects of feather-fiber reinforcement on poly(vinyl alcohol)/clay aerogels: Structure, property and applications. Polymer.

[23] Pourjavaheri F., Mohades F., Jones O., Sherkat F., Kong I., Gupta A., Shanks R.A. (2017). Avian keratin fibre-based bio-composites. World J. Eng..

[24] Wang J., Hao S., Luo T., Yang Q., Wang B. (2016). Development of feather keratin nanoparticles and investigation of their hemostatic efficacy. Mater. Sci. Eng. C.

[25] Wang J., Hao S., Luo T., Cheng Z., Li W., Gao F., Guo T., Gong Y., Wang B. (2017). Feather keratin hydrogel for wound repair: Preparation, healing effect and biocompatibility evaluation. Colloids Surfaces B Biointerfaces.

[26] Thonpho A., Srihanam P. (2016). Preparation and Characterization of Keratin Blended Films using Biopolymers for Drug Controlled Release Application. Orient. J. Chem..

[27] Pajarito B.B., Belarmino A.J., Calimbas R.M., Gonzales J.R. (2020). Graphite Nanoplatelets from Waste Chicken Feathers. Materials.

[28] Asdrubali F., D’Alessandro F., Schiavoni S. (2015). A review of unconventional sustainable building insulation materials. Sustain. Mater. Technol..

[29] Acda M.N. (2010). Waste chicken feather as reinforcement in cement-bonded composites. Philipp. J. Sci..

[30] Winandy J.E., Muehl J.H., Micales J.A., Raina A., Schmidt W. Potential of Chicken Feather Fibre in Wood MDF Composites. Proceedings of the 2nd International Conference on EcoComposites – EcoComp.

[31] Taghiyari H.R., Majidi R., Esmailpour A., Samadi Y.S., Jahangiri A., Papadopoulos A.N. (2020). Engineering Composites Made from Wood and Chicken Feather Bonded with UF Resin Fortified with Wollastonite: A Novel Approach. Polymers.

[32] Bessa J., Souza J., Lopes J., Sampaio J., Mota C., Cunha F., Fangueiro R. (2017). Characterization of thermal and acoustic insulation of chicken feather reinforced composites. Procedia Eng..

[33] Strnad S., Kreže T. Poultry feather wastes - optimization of cleaning procedure for new products development. Proceedings of the International Conference on Innovative Technologies, IN-TECH 2011.

[34] Bratina B., Šorgo A., Kramberger J., Ajdnik U., Zemljič L.F., Ekart J., Šafarič R. (2016). From municipal/industrial wastewater sludge and FOG to fertilizer: A proposal for economic sustainable sludge management. J. Environ. Manag..

[35] ISO 10534 (1998). Acoustics—Determination of Sound Absorption Coefficient and Impedance in Impedance Tubes.

[36] ASTM E1050-90 (2008). Standard Test Method for Impedance and Absorption of Acoustical Materials Using a Tube, Two Microphones and a Digital Frequency Analysis System.

[37] ASTM C384-98 (1998). Standard Test Method for Impedance and Absorption of Acoustical Materials by the Impedance Tube Method.

[38] Selamet A., Ji Z. (1999). Acoustic attenuation performance of circular expansion chambers with extended inlet/outlet. J. Sound Vib..

[39] Lee Y., Ng C. (1998). Sound insertion loss of stiffened enclosure plates using finite element method and the classical approach. J. Sound Vib..

[40] Karlinasari L., Hermawan D., Maddu A., Bagus M., Khrisna Lucky I., Nugroho N., Sudo Hadi Y. (2012). Acoustical Properties of particleboards made from betung bamboo (Dendrocalamus asper) as building construction material. Biol. Res..

