# Supplementary material for: Preparation and Characterisation of Waste Poultry Feathers Composite Fibreboards
Source: Materials (Basel). 2020 Nov 4;13(21):4964. doi: 10.3390/ma13214964 (PMC7663731; doi:10.3390/ma13214964)
Supplement: Supplementary file 1 [file materials-13-04964-s001.pdf]

materials

Supplementary Materials: Preparation and Characterisation of Waste Poultry Feathers Composite Fibreboards

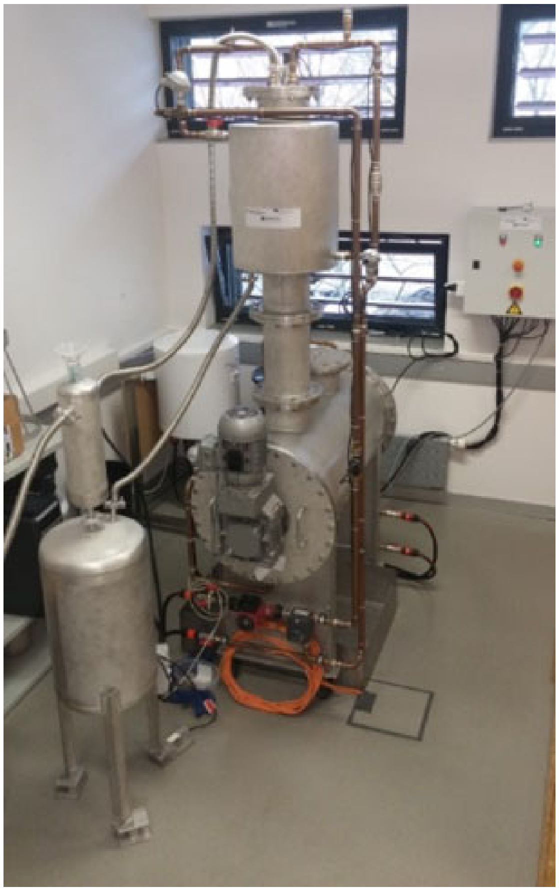

Figure S1: The vacuum drier.

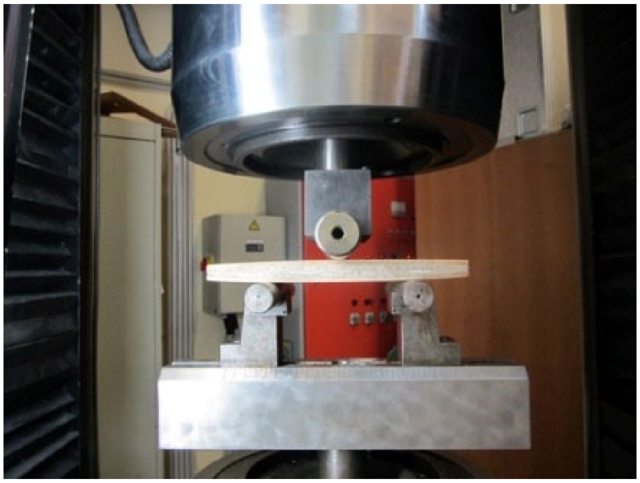

Figure S2: The three-point bending test performance using the servo-hydraulic testing machine INSTRON 1255.
